# Latent class analysis of the capacity of countries to manage diabetes and its relationship with diabetes-related deaths and healthcare costs

**DOI:** 10.1186/s12913-024-12052-2

**Published:** 2025-01-15

**Authors:** Samuel Akyirem, Emmanuel Ekpor, Charles Boakye Kwanin

**Affiliations:** 1 School of Nursing, Yale University, Orange, CT USA; 2 School of Nursing and Midwifery, University of Ghana, Accra, Ghana; 3 School of Nursing, University of Washington, Seattle, WA USA

**Keywords:** Diabetes, Diabetes-related death, Diabetes-related expenditure, Latent class analysis, National capacity

## Abstract

**Background:**

The prevalence of diabetes is escalating globally, underscoring the need for comprehensive evidence to inform health systems in effectively addressing this epidemic. The purpose of this study was to examine the patterns of countries’ capacity to manage diabetes using latent class analysis (LCA) and to determine whether the patterns are associated with diabetes-related deaths and healthcare costs.

**Methods:**

Eight indicators of country-level capacity were drawn from the World Health Organization Global Health Observatory dataset: the widespread availability of hemoglobin A1C (HbA1c) testing, existence of diabetes registry, national diabetes management guidelines, national strategy for diabetes care, blood glucose testing, diabetic retinopathy screening, sulfonylureas, and metformin in the public health sector. We performed LCA of these indicators, testing 1–5 class solutions, and selecting the best model based on Bayesian Information Criteria (BIC), entropy, corrected Akaike Information Criteria (cAIC), as well as theoretical interpretability. Multivariable linear regression was used to assess the association between capacity to manage diabetes (based on the latent class a country belongs) and diabetes-related deaths and healthcare costs.

**Results:**

We included 194 countries in this secondary analysis. Countries were classified into “high capacity” (88.7%) and “limited capacity” (11.3%) countries based on the two-class solution of the LCA (entropy = 0.91, cAIC = 1895.93, BIC = 1862.93). Limited capacity countries were mostly in Africa. Limited capacity countries had significantly higher percentage of their deaths attributable to diabetes (adjusted beta = 1.34; 95% CI: 0.15, 2.53; *p* = 0.027) compared to high capacity countries even after adjusting for income status and diabetes prevalence.

**Conclusions:**

Our findings support the report by the Lancet commission on diabetes, which suggests that differences in diabetes outcomes among countries may be explained by variations in the capacity of and investments made in their health systems. Future studies should evaluate initiatives such as the WHO Global Diabetes Compact that are currently underway to improve the capacity of resource-limited countries.

**Supplementary Information:**

The online version contains supplementary material available at 10.1186/s12913-024-12052-2.

## Introduction

Diabetes is a pressing global health concern, affecting 537 million adults in the year 2021 [1]. The International Diabetes Federation projects that diabetes will affect 783 million people worldwide by the year 2045 (representing a 46% rise in the prevalence rate recorded in 2021). Diabetes imposes a significant financial strain on healthcare systems, resulting in health expenditures of US$966 billion globally in 2021 [1]. In addition to the economic burden, individuals grappling with diabetes experience a diminished health-related quality of life [2], have a 2–4 times increased cardiovascular risk [3], and a 2–3 folds risk of all-cause mortality [4]. Recent data from the Global Burden of Disease (GBD) study reveal that diabetes ranked among the top 10 cause of death, claiming over 1.6 million lives globally in 2021 [5].

Despite the global ubiquity of diabetes, there is significant variations in its burden across nations, with approximately 80% of diagnosed cases clustered in low- and middle-income countries (LMICs) [1]. Moreover, previous study has shown that LMICs account for majority of diabetes-related disability and mortality cases [6]. The Lancet Commission on diabetes reports that this disparity is partly attributed to differing national capacities to manage the disease, underscoring the importance of understanding such variability to devise effective public health interventions and healthcare policies tailored to each country’s unique needs [7]. The World Health Organization (WHO) delineates various indicators such as the availability of treatment guidelines, action plan, registries, and other factors in assessing national capacities to manage diabetes and other noncommunicable diseases (NCDs) [8]. Yet, a notable gap persists in the literature regarding comprehensive assessments of the capacity of various countries to manage diabetes. Previous studies on this topic are centered on specific regions, and often focus on specific aspects of diabetes care, without fully capturing the multidimensional nature of national capacity indicators [9, 10]. Moreover, whiles it is plausible that the capacity of countries to manage diabetes could be associated with care outcome including diabetes-related deaths and healthcare costs, no study has integrated these factors to provide a holistic understanding of this relationship.

The purpose of this study was to examine the patterns of countries’ capacity to manage diabetes using latent class analysis and to determine whether the patterns are associated with diabetes-related deaths and healthcare costs. Latent class analysis is a multivariate statistical technique which uses observed indicators to identify latent (unobserved) groups that are present within a population [11]. By applying LCA, we can categorize countries into distinct classes based on multiple indicators used to assess country-level diabetes management capacity. Additionally, by investigating how a country’s capacity to manage diabetes relates to their diabetes deaths and cost, we hope to highlight the need for governments to adequately resource and invest in healthcare systems. The findings from this study may allow stakeholders including WHO to provide targeted support to member States that have low capacity to manage diabetes.

## Method

### Study design

We conducted a cross-sectional analysis of secondary data obtained from the World Health Organization (WHO), International Diabetes Federation (IDF), and the World Bank Group to identify different patterns of country-level capacities to manage diabetes and how these patterns are associated with diabetes-specific healthcare expenditure and deaths. Countries were eligible for inclusion if they participated in the 2021 WHO NCD country capacity survey.

### Measures and data sources

#### Predictor

##### Capacity to manage diabetes

Country-level capacity to manage diabetes was assessed using eight indicators from the WHO Global Health Observatory (GHO) dataset which contains official country responses to the WHO NCD country capacity survey [12]. The NCD country capacity survey is a periodic assessment conducted by the WHO to enable countries to assess the progress made in improving their capacity to address common NCDs. From May to June 2021, the WHO contacted designated individuals or officials responsible for NCDs in each of the 194 member countries to complete an online NCD country capacity survey. In completing the survey, the designated individuals were required to attach supporting evidence to some questions (e.g. diabetes care guidelines or policy document, if a country indicates that they have a policy or guidelines on diabetes care) to enable WHO to validate countries’ responses to survey questions. WHO then reviewed all survey responses and compared responses to existing data to establish veracity and consistency. The WHO also contacted countries for clarifications and additional supporting documentations on their responses as needed [12].

The eight indicators drawn from the NCD country capacity survey and included in the current analysis are: (1) general availability of diabetes testing (by HbA1c) at the primary health care level, (2) existence of diabetes registry, (3) existence of evidence-based national guidelines/protocols/standards for the management of diabetes, (4) existence of operational policy/strategy/action plan for diabetes, (5) general availability of diabetes testing (by blood glucose measurement, OGTT) at the primary health care level, (6) general availability of diabetic retinopathy screening in the public health system, (7) general availability of sulfonylureas in the public health sector, and (8) general availability of metformin in the public health sector. These indicators were selected because they reflect the multidimensional nature of countries’ capacity to manage diabetes, including the availability of essential diagnostic tools and medications and the presence of structured policies and guidelines. Official responses to the questions included “yes”, “no”, “no response”, or “don’t know”. All “no response” and “don’t know” responses were recoded into a third category (“data not available”) instead of treating them as missing data. This approach was based on the rationale that a country’s ability to accurately report such critical health metrics may be indicative of the overall strength and robustness of its health infrastructure, hence treating them as missing data may result in loss of valuable data.

#### Outcome variables

##### Diabetes-related deaths

We abstracted mortality data from the IDF dashboard for each country [13]. Diabetes-related deaths were measured as the percentage of deaths among individuals under 60 years of age that is attributable to diabetes.

##### Diabetes-related healthcare cost

Country-level diabetes-related healthcare costs were normalized by dividing the diabetes-related health expenditure per person by the per capita health expenditure for the country. This metric was used in lieu of raw diabetes-related health expenditure, to allow for comparison across countries with different levels of health spending and economic statuses. Data for diabetes-related health expenditure per person was abstracted from the IDF dashboard in United States Dollars [14]. Similarly, per capita health expenditure for each country was obtained from the WHO GHO dataset [12].

#### Covariates

##### Diabetes prevalence

The 2021 age-adjusted comparative prevalence of diabetes among adults aged from 20 to 79 years was obtained from the IDF dashboard country [13]. Prevalence was expressed as a percentage of the adult population.

##### World Bank country classification by income level

Country classifications by income level were obtained from the World Bank [15]. Country classifications were recoded as “high income” vs. “low-middle income” to ensure adequate sample size within each category to support robust regression analysis.

### Analysis

Data were analyzed in R programming language. We performed latent class analysis of the eight indicators of countries’ capacity to manage diabetes using the “polCA” package in R [16]. We run 1 to 5 class solutions for the latent class analysis and selected the most appropriate model using both statistical and theoretical interpretability criteria. The statistical criteria for model selection included the most reliable indicator of model fit, Bayesian Information Criteria (BIC; lower values preferred) [17] as well as other fit indices such as likelihood ratio (lower values preferred), sample-size adjusted BIC (aBIC; lower values preferred), corrected Akaike Information Criteria (cAIC; lower values preferred), and entropy (> 0.70 is acceptable). After identifying the most appropriate class solution, we labelled each class by examining the conditional probabilities of item responses in each class. Next, we used multivariable linear regression to assess the association between capacity to manage diabetes (based on the latent class a country belongs) and diabetes-related deaths and healthcare cost while adjusting for countries’ diabetes prevalence and income classifications. For the regression analysis, missing data in diabetes prevalence, deaths, and expenditure variables were handled by listwise deletion given the low missingness in these variables (1%, 5.7%, 4.6%, and 6.2% of the sample had missing diabetes prevalence, diabetes-related deaths, income classification, and healthcare cost data respectively). Overall, 18 (9.3%) countries were excluded from the “death model” and 19 (9.8%) countries from the “healthcare cost model” (supplementary file 2). For the “death model”, countries that were excluded did not have significantly different geographical distribution and capacity to manage diabetes compared to those that had complete data and were retained in the model (supplementary file 2). However, for the healthcare cost model, 6/47 countries in Europe and 9/18 countries in the West Pacific were excluded. Countries that were excluded from this model had significantly different geographical distribution from those that were retained in this model (*p* = 0.0001). A p-value of 5% or less was considered statistically significant.

## Findings

### Characteristics of study sample

A total of 194 countries had data on the 8 indicators selected to access countries’ capacity to manage diabetes and thus formed the analytic sample. As shown in Table 1, majority of included countries were in Europe (*N* = 53, 27.32%) followed by Africa (*N* = 47, 24.23%). Majority of the countries were low-middle income (*N* = 128, 65.98%). Pakistan had the highest age-adjusted diabetes prevalence of 30.8% with 17.5% of the country’s mortality attributed to diabetes. Zimbabwe had the highest diabetes-related healthcare cost, spending more than 12 times as much on diabetes care relative to its per capita healthcare expenditure.

**Table 1:** Characteristics of study sample (*N* = 194)

Characteristics	*N* (%)
World Bank classification	
Low and middle income	128 (65.98)
High income	57 (29.38)
Missing	9 (4.64)
Region	
Africa	47 (24.23)
Americas	35 (18.04)
Eastern Mediterranean	21 (10.82)
Europe	53 (27.32)
South-East Asia	11 (5.67)
Western Pacific	27 (13.92)
Existence of diabetes registry	
Yes	89 (45.87)
No	98 (50.52)
Data not available	7 (3.61)
General availability of diabetes testing at the primary health care level	
Yes	179 (92.27)
No	7 (3.61)
Data not available	8 (4.12)
Has evidence-based national guidelines/protocols/standards for the management of diabetes	
Yes	173 (89.17)
No	20 (10.31)
Data not available	1 (0.52)
Has diabetic retinopathy screening in the public health system	
Yes	132 (68.04)
No	48 (24.74)
Data not available	14 (7.22)
General availability of metformin in the public health sector	
Yes	174 (89.69)
No	13 (6.70)
Data not available	7 (3.61)
General availability of sulfonylureas in the public health sector	
Yes	148 (76.29)
No	34 (17.52)
Data not available	12 (6.19)
General availability of diabetes testing (by HbA1c) at the primary health care level	
Yes	136 (70.10)
No	37 (19.07)
Data not available	21 (10.83)
Existence of operational policy/strategy/action plan for diabetes	
Yes	134 (69.07)
No	59 (30.41)
Data not available	1 (0.52)

### Latent class analysis

Table 2 shows the results of the 1 to 5 latent class models and their respective fit indices. We determined that the two-class solution is the most appropriate model because it had the lowest BIC ( 1862.93), cAIC (1895.93), and the highest entropy (0.91), as well as being easily interpretable. The majority of countries were in class 2 (*N* = 172, 88.7%) with class 1 containing only 11.3% (*N* = 22) of the sample.

**Table 2:** Fit statistics for 1 to 5 class solutions

Model	Number of classes	Likelihood ratio	cAIC	BIC	aBIC	Entropy	Smallest class %
1	1	546.12	1978.81	1962.81	1912.13	-	-
**2**	**2**	**356.68**	**1895.93**	**1862.93**	**1758.39**	**0.91**	**11.34%**
3	3	285.34	1931.14	1881.14	1722.75	0.88	3.61%
4	4	235.62	1987.98	1920.98	1708.74	0.85	3.61%
5	5	211.85	2070.75	1986.75	1720.66	0.76	4.12%

Countries in latent class 1 were less likely to have diabetes registry, had limited access to sulfonylureas and metformin which are basic diabetes medication, and limited access to HbA1C testing which is essential for monitoring the progress of diabetes management. This class also had limited availability of retinopathy screening in the public health system. Countries in this group, however, had high probabilities of having diabetes treatment protocols and national diabetes strategic plans. Additionally, countries in this class had high probability of not having data on multiple indicators of capacity to manage diabetes including whether or not A1C testing is widely available at primary healthcare level as shown in Table 2. We labelled this class as “limited capacity” countries.

**Fig. 1:**
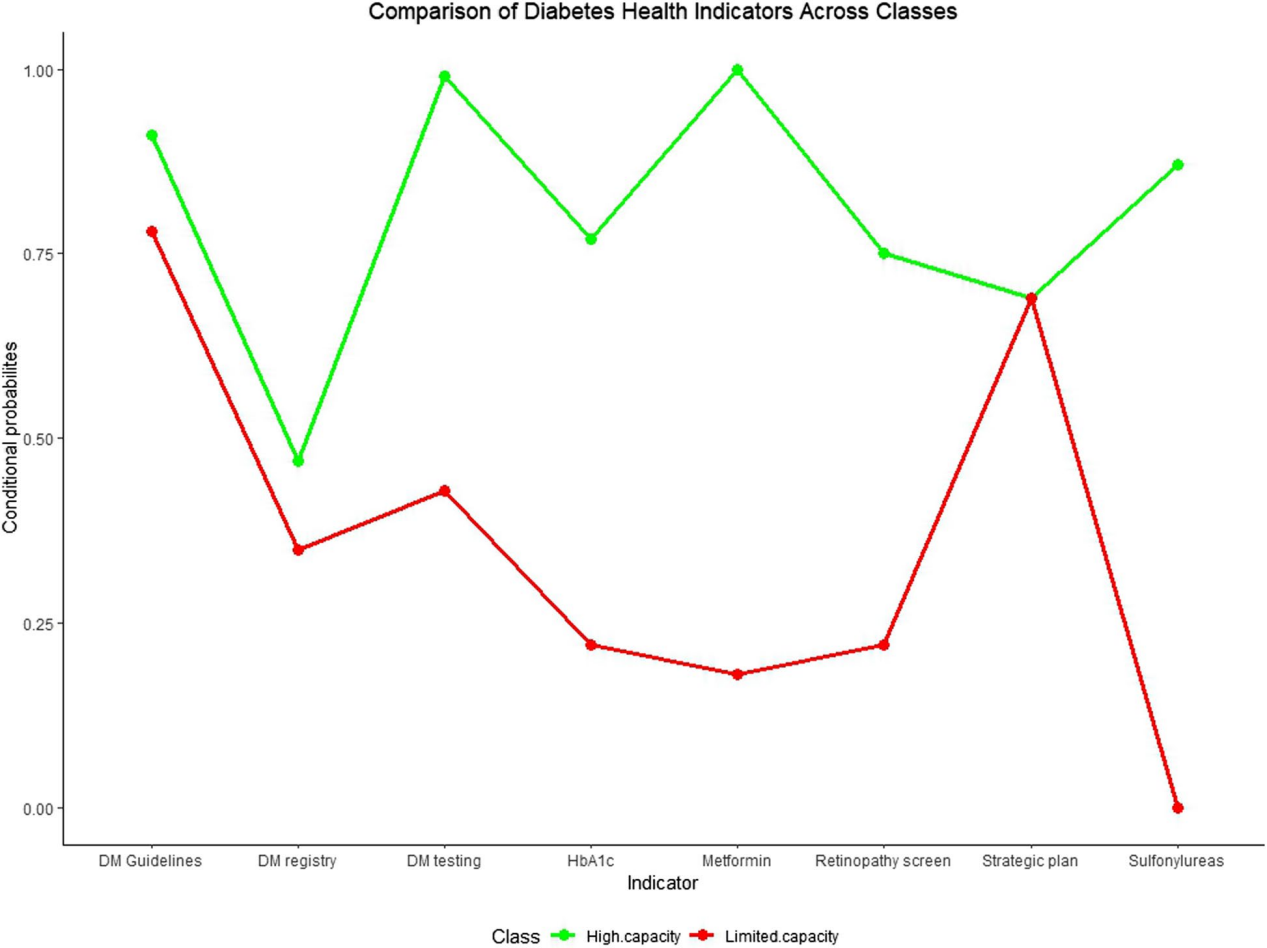
Plot of conditional probabilities of "yes" responses by latent classes

Latent class 2 included countries that had a high probability of having diabetes registry, widespread availability of diabetes testing including HbA1C, availability of essential diabetes medications, and existence of diabetes strategic plan and protocols for diabetes management as shown in Table 3; Fig. 1. Countries in this class had very low probability of not having data on any of the indicators used to assess countries’ capacity to manage diabetes. We therefore labelled class 1 as “high capacity” countries.

**Table 3:** Conditional probabilities of the item responses for the 2-class solution

	Class 1–11.3%			Class 2–88.7%		
	Yes	No	Data not available	Yes	No	Data not available
Existence of diabetes registry	0.35	0.49	0.16	0.47	0.51	0.02
General availability of diabetes testing at the primary health care level	0.43	0.24	0.33	0.99	0.01	0.00
Has evidence-based national guidelines/protocols/standards for the management of diabetes	0.78	0.22	0.00	0.91	0.09	0.00
Has diabetic retinopathy screening in the public health system	0.22	0.49	0.29	0.75	0.21	0.04
General availability of metformin in the public health sector	0.18	0.53	0.29	1.00	0.00	0.00
General availability of sulfonylureas in the public health sector	0.00	0.73	0.27	0.87	0.10	0.03
General availability of diabetes testing (by HbA1c) at the primary health care level	0.22	0.43	0.35	0.77	0.16	0.07
Existence of operational policy/strategy/action plan for diabetes	0.69	0.27	0.04	0.69	0.31	0.00

**Fig. 2:**
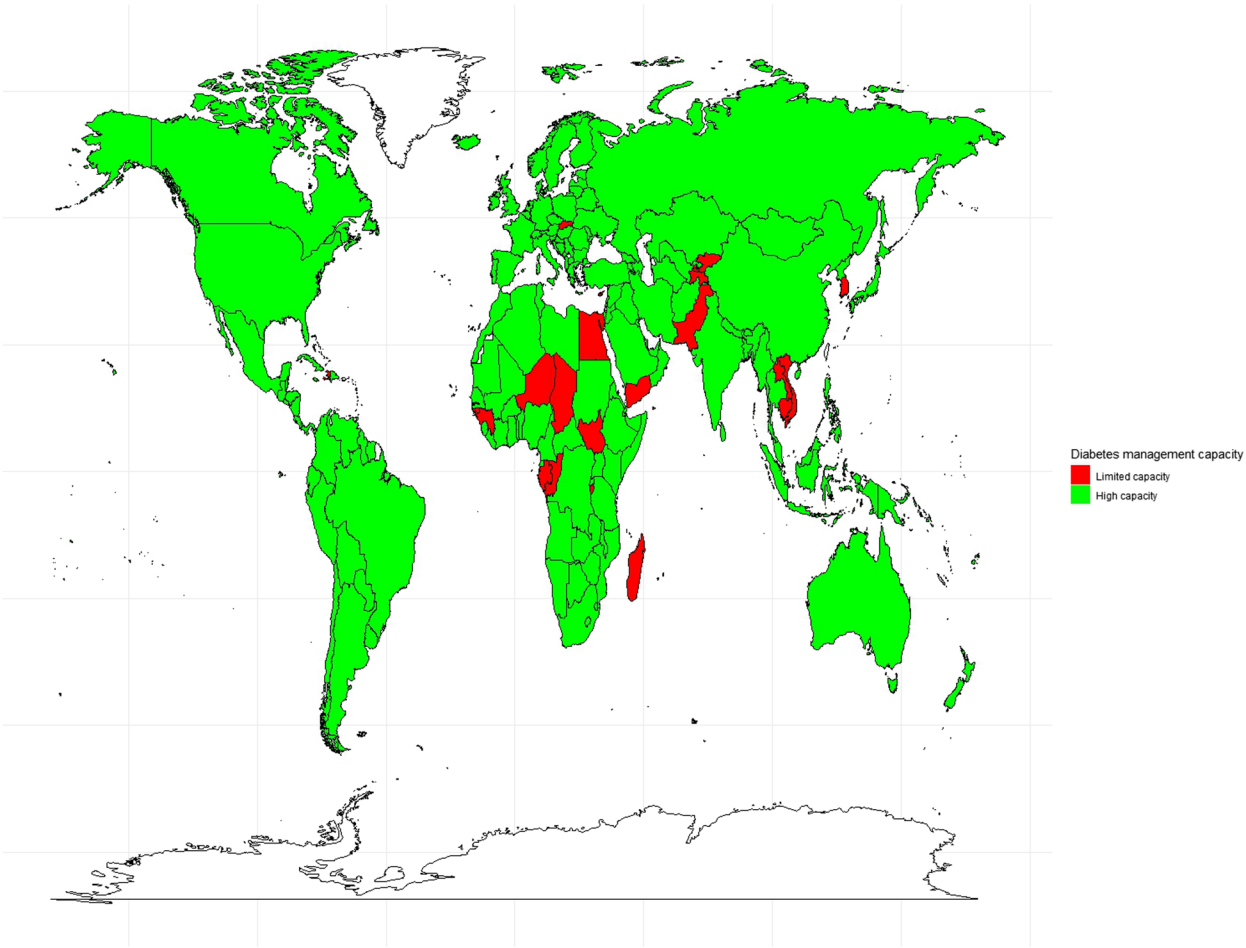
Heat map showing the national capacity of countries to manage diabetes

As shown in Fig. 2, most of the 22 “limited capacity” countries were in Africa (*N* = 10; representing 45.45% of the African countries included in this study). Countries in North America, Europe, Australia often belonged to the “high capacity” group.

#### Results of the multivariable regression

Table 4 shows the results of the two multivariable linear regression models. Countries in limited capacity group had significantly higher percentage of their deaths attributable to diabetes even after adjusting for income status and diabetes prevalence (adjusted beta = 1.34; 95% CI: 0.15, 2.53; *p* = 0.027). There was no significant association between a country’s latent class and diabetes-related healthcare costs after adjusting for diabetes prevalence and countries’ income classification.

**Table 4:** Association between a country’s capacity to manage diabetes and diabetes-related healthcare expenditure and deaths

	Diabetes-related healthcare costs		Diabetes-related deaths.	
Variables	Adjusted Beta (95% CI)	p-value	Adjusted Beta (95% CI)	p-value
Capacity to manage diabetes				
High capacity	[Reference]		[Reference]	
Limited capacity	0.41 (−0.35, 1.18)	0.288	1.34 (0.15, 2.53)	0.027
Prevalence	−0.10 (−0.14, −0.06)	< 0.0001	0.54 (0.47, 0.61)	< 0.0001
WB classification				
High income	[Reference]		[Reference]	
LMIC	2.15 (1.70, 2.59)	< 0.0001	1.55 (0.79, 2.32)	< 0.0001
Model fit statistics	Adjusted *R*^*2*^: 41.21%;$\:{F}_{3,\:171}$= 41.65, *p* < 0.0001		Adjusted *R*^*2*^: 57.71%;$\:{F}_{3,\:172}$= 80.6, *p* < 0.0001	

## Discussion

The current study is the first to assess the capacity of countries to manage diabetes using multiple national indicators concurrently. Using latent class analysis, countries were classified into “high capacity” and “limited capacity” countries. Our results indicate that countries with limited capacity to manage diabetes were mostly found in the continent of Africa. We observed higher diabetes-related deaths among countries with limited capacity to manage diabetes. Our findings affirm the report by the Lancet Commission on Diabetes that differences in diabetes outcomes among countries may be explained by the differences in the capacity of and investments made in their health systems [7]. These findings highlight the critical need for strengthening health system capacity to better manage diabetes, improve health outcomes, and reduce the associated mortality burden.

The two clusters of countries identified in this study demonstrated considerable differences in their widespread availability of diabetes registry, HbA1C testing, and retinopathy screening. Diabetes registries enable countries to monitor and evaluate their diabetes care quality to inform policy changes and healthcare financing decisions [18]. Diabetes registries also allow countries to track and manage high-risk patients to prevent complications [19]. Similarly, HbA1C testing is essential for diagnosing diabetes and assessing glycemic control for persons with diabetes. The widespread availability of this test enables countries to provide timely diagnosis, treatment, and monitoring of glycemic control to inform adjustments to medications and prevent complications [20]. Lastly, the availability of retinopathy screening may be indicative of the general attention to the assessment of microvascular complications of diabetes in these countries. Retinopathy is considered one of the earliest vascular changes that occur with diabetes, and early identification can prevent further microvascular and macrovascular complications occurring in other parts of the body [21]. By having widespread availability to these resources, high-capacity countries compared to limited-capacity countries, are better placed to provide high quality diabetes care that is associated with better health outcomes and reduced diabetes-related mortality [9].

Our findings also highlight the opportunity cost associated with limited investment in health systems to support diabetes management. Although not statistically significant, we observed that countries with limited capacity had higher diabetes-related healthcare expenditure. Limited capacity to manage diabetes, as characterized by poor availability of essential diabetes tracking (via registry), testing, and medications may be associated with higher rates of hospitalization, diabetes complications, and the need for intensive treatment – all of which contribute to higher diabetes-related health expenditure [7]. In countries (e.g. African countries), where there is limited universal health and national health insurance coverage, patients may have to bear this high diabetes-related health expenditure themselves, further worsening the financial burden on patients and their families [22]. This financial burden is associated with reduced access to essential care, as patients might forego treatment due to cost, resulting in worse diabetes outcomes [23] – thus potentially creating a vicious cycle of high cost and poor diabetes outcomes. It was thus not surprising that even after adjusting for diabetes prevalence, we observed a significant association between limited capacity to manage diabetes and deaths attributable to diabetes.

The finding that most limited capacity countries were in Africa may be indicative of the general lack of recognition by governments and policy makers of the epidemiological transition from infectious diseases to NCDs been witnessed in the continent. Countries in Africa often contend with high burden of communicable diseases and maternal health challenges that take considerable amount of the countries’ healthcare financing budget, leaving limited space for the emerging diabetes epidemic [24]. The need to make significant investment in health systems to support diabetes care has been highlighted in previous studies. For instance, a large cross-sectional study of 847,413 individuals across 28 LMICs, reported health systems limitations that resulted in only 38.4% of persons with diabetes receiving treatment (medication and/or education on lifestyle modification) [10]. Similarly, a systematic review of the readiness of healthcare systems in African countries to manage diabetes found that across most countries in the continent, there was limited availability of diabetes diagnostic services, essential medications, electronic medical record systems, and diabetes health professionals [25]. It is therefore essential for future interventions to target these limited capacity regions or countries.

Some initiatives have been launched to improve the capacity of resource-limited countries to manage diabetes. In 2021, the WHO launched the Global Diabetes Compact (GDC) to reduce inequities in global access to diabetes diagnosis and treatment services [26]. The program aims to support African countries and other LMICs to strengthen their health systems to implement evidence-based interventions based on country-specific needs [27]. This program is still in its infancy and future analyses should examine changes in the capacity of countries to manage diabetes following the implementation of the GDC strategies. Other approaches to improve capacity may emphasize the integration of diabetes management into existing and well-established infectious diseases management platforms especially in LMICs. Several studies in sub-Saharan Africa have demonstrated that integration of diabetes care into existing HIV or tuberculosis management platforms can limit competition between infectious and non-infectious disease for resources and can present as a low-cost option to improve the capacity of countries to manage diabetes by building on existing structures [7, 25]. Additionally, governments in limited capacity countries should adequately invest in healthcare systems and enact and enforce diabetes-related or more broadly, NCD-related policies to support diabetes management. Policies and legislation may help direct governmental fund allocations to develop comprehensive diabetes management programs, improve healthcare infrastructure, and ensure the availability of essential medications and diagnostic tools at the primary healthcare level. Such investments can also enhance healthcare workforce training and support public health initiatives for diabetes prevention and education. These solutions may not be a one size fits all as there is considerable diversity in healthcare systems globally. Future studies should also examine contextual factors including political will, that can inform local policies and interventions on improving capacity to manage diabetes.

The current analysis has several limitations. First, it is likely that some country representatives misinterpreted questions from the WHO NCD Capacity survey which assessed, among other things, the capacity of countries to manage diabetes. Moreover, the focus of the questions on the public health sector may mean that for countries that significantly rely on the private health sector, their responses may not reflect the true status of their capacity to manage diabetes. Second, there may be significant in-country heterogeneity in terms of availability of resources to manage diabetes which we could not evaluate. Third, while our analysis focused on availability of essential diabetes resources, the analysis could have been strengthened by incorporating a metric to determine the affordability of these resources. Such metric may include, for instance, whether HbA1C testing or essential medicines are covered by the common or basic health insurance policies in the country. Additionally, there are important potentially confounding variables for which data were not readily available. These variables include country corruption indices, political stability, human right violation, aged population, cost of living, and presence of social safety net programs. The impact of these variables on the associations tested in this study should be explored in future research. Fourth, given the cross-sectional design of this study, we are unable to make causal conclusions regarding the relationship between countries’ capacity to manage diabetes and diabetes-related outcomes. Future studies should adopt longitudinal study design to evaluate how changes in national capacity impact diabetes outcomes in the medium- and long-terms. Fifth, while the IDF is a reputable source of diabetes data, its diabetes prevalence estimates for LMICs are less reliable due to the lack of in-country data for about one-third of countries, particularly in Africa, leading to reliance on extrapolations from neighboring countries [28]. The unreliable prevalence estimates for LMICs may be a symptom of a bigger problem, i.e., deficiencies in health infrastructure and limited investments to enhance capacity to manage or track diabetes in those countries. These unreliable estimates may thus bias the associations tested in this study. We, however, mitigated the bias by adjusting the regression models for country-level income classifications (LMICs vs. high income).

## Conclusions

There are significant disparities in national capacities to address diabetes. Countries with limited capacity, predominantly located in Africa are more likely to experience greater diabetes-related mortality rates. Despite the limitations of this study, it underscores the urgent need for targeted interventions and international support to bolster the diabetes management infrastructure in lower-capacity countries.

## Supplementary Information

Supplementary Material 1.

Supplementary Material 2.

## Data Availability

The datasets generated and/or analyzed during the current study can be accessed through the references provided in this published article (see “methods”).
