# Supplementary material for: Latent class analysis of the capacity of countries to manage diabetes and its relationship with diabetes-related deaths and healthcare costs
Source: BMC Health Serv Res. 2025 Jan 15;25:83. doi: 10.1186/s12913-024-12052-2 (PMC11734536; doi:10.1186/s12913-024-12052-2)
Supplement: Supplementary file 1 — Supplementary Material 1. [file 12913_2024_12052_MOESM1_ESM.docx]

**List of limited capacity countries (n=22)**

1. Burundi
2. Cambodia
3. Chad
4. Republic of Congo
5. Cyprus
6. Egypt
7. Gabon
8. Guinea
9. Guinea-Bissau
10. Haiti
11. Kyrgyzstan
12. Laos
13. Lesotho
14. Madagascar
15. Niger
16. Pakistan
17. South Korea
18. Slovakia
19. South Sudan
20. Tajikistan
21. Vietnam
22. Yemen
