# Supplementary material for: Latent class analysis of the capacity of countries to manage diabetes and its relationship with diabetes-related deaths and healthcare costs
Source: BMC Health Serv Res. 2025 Jan 15;25:83. doi: 10.1186/s12913-024-12052-2 (PMC11734536; doi:10.1186/s12913-024-12052-2)
Supplement: Supplementary file 2 — Supplementary Material 2. [file 12913_2024_12052_MOESM2_ESM.docx]

**Supplementary material 2**

**S1. Countries included or excluded from the “healthcare cost model” due to missingness in one or more of the variables included in the model**

| **Excluded** | **Included** |
| --- | --- |
| 1. Bolivia 2. Cook Islands 3. Cuba 4. North Korea 5. Egypt 6. Equatorial Guinea 7. Kyrgyzstan 8. Lao 9. Libya 10. Montenegro 11. Niue 12. South Korea 13. Moldova 14. Slovakia 15. Somalia 16. South Sudan 17. Syrian 18. Vietnam 19. Yemen | 1. Côte d’Ivoire 2. Afghanistan 3. Albania 4. Algeria 5. Andorra 6. Angola 7. Antigua and Barbuda 8. Argentina 9. Armenia 10. Australia 11. Austria 12. Azerbaijan 13. Bahamas 14. Bahrain 15. Bangladesh 16. Barbados 17. Belarus 18. Belgium 19. Belize 20. Benin 21. Bhutan 22. Bosnia and Herzegovina 23. Botswana 24. Brazil 25. Brunei Darussalam 26. Bulgaria 27. Burkina Faso 28. Burundi 29. Cabo Verde 30. Cambodia 31. Cameroon 32. Canada 33. Central African Republic 34. Chad 35. Chile 36. China 37. Colombia 38. Comoros 39. Congo 40. Costa Rica 41. Croatia 42. Cyprus 43. Czechia 44. Democratic Republic of the Congo 45. Denmark 46. Djibouti 47. Dominica 48. Dominican Republic 49. Ecuador 50. El Salvador 51. Eritrea 52. Estonia 53. Eswatini 54. Ethiopia 55. Fiji 56. Finland 57. France 58. Gabon 59. Gambia 60. Georgia 61. Germany 62. Ghana 63. Greece 64. Grenada 65. Guatemala 66. Guinea 67. Guinea-Bissau 68. Guyana 69. Haiti 70. Honduras 71. Hungary 72. Iceland 73. India 74. Indonesia 75. Iran (Islamic Republic of) 76. Iraq 77. Ireland 78. Israel 79. Italy 80. Jamaica 81. Japan 82. Jordan 83. Kazakhstan 84. Kenya 85. Kiribati 86. Kuwait 87. Latvia 88. Lebanon 89. Lesotho 90. Liberia 91. Lithuania 92. Luxembourg 93. Madagascar 94. Malawi 95. Malaysia 96. Maldives 97. Mali 98. Malta 99. Marshall Islands 100. Mauritania 101. Mauritius 102. Mexico 103. Micronesia (Federated States of) 104. Monaco 105. Mongolia 106. Morocco 107. Mozambique 108. Myanmar 109. Namibia 110. Nauru 111. Nepal 112. Netherlands 113. New Zealand 114. Nicaragua 115. Niger 116. Nigeria 117. North Macedonia 118. Norway 119. Oman 120. Pakistan 121. Palau 122. Panama 123. Papua New Guinea 124. Paraguay 125. Peru 126. Philippines 127. Poland 128. Portugal 129. Qatar 130. Romania 131. Russian Federation 132. Rwanda 133. Saint Kitts and Nevis 134. Saint Lucia 135. Saint Vincent and the Grenadines 136. Samoa 137. San Marino 138. Sao Tome and Principe 139. Saudi Arabia 140. Senegal 141. Serbia 142. Seychelles 143. Sierra Leone 144. Singapore 145. Slovenia 146. Solomon Islands 147. South Africa 148. Spain 149. Sri Lanka 150. Sudan 151. Suriname 152. Sweden 153. Switzerland 154. Tajikistan 155. Thailand 156. Timor-Leste 157. Togo 158. Tonga 159. Trinidad and Tobago 160. Tunisia 161. Turkey 162. Turkmenistan 163. Tuvalu 164. Uganda 165. Ukraine 166. United Arab Emirates 167. United Kingdom of Great Britain and Northern Ireland 168. United Republic of Tanzania 169. United States of America 170. Uruguay 171. Uzbekistan 172. Vanuatu 173. Venezuela (Bolivarian Republic of) 174. Zambia 175. Zimbabwe |

**S2. Comparing the regional distribution of the 19 countries excluded from the “healthcare cost model” with those included in the model**

|  | Africa | Americas | Eastern Mediterranean | Europe | South-East Asia | Western Pacific |
| --- | --- | --- | --- | --- | --- | --- |
| Excluded from the model (n, %) | 2 (4.3) | 2 (5.7) | 5 (23.8) | 4 (7.5) | 1 (9.1) | 5 (18.5) |
| Included in the model (n, %) | 45 (95.7) | 33 (94.3) | 16 (76.2) | 49 (92.5) | 10 (90.1) | 22 (81.5) |

P = 0.088, *χ*2=9.596

**S3. Comparing the capacity of the 19 countries excluded from the “healthcare cost model” to manage diabetes with those included in the model**

|  | High capacity | Low capacity |
| --- | --- | --- |
| Excluded from the model (n, %) | 15 (8.8) | 4 (18.2) |
| Included in the model (n, %) | 156 (91.2) | 18 (81.8) |

P = 0.163, χ2=1.945

**S4. Countries included or excluded from the “death model” due to missingness in one or more of the variables included in the model**

| **Excluded** | **Included** |
| --- | --- |
| 1. Andorra 2. Bolivia 3. Cook Islands 4. Dominica 5. Kyrgyzstan 6. Laos 7. Marshall Islands 8. Monaco 9. Nauru 10. Niue 11. Palau 12. South Korea 13. Moldova 14. Saint Kitts and Nevis 15. San Marino 16. Slovakia 17. Tuvalu 18. Vietnam | 1. Côte d’Ivoire 2. Afghanistan 3. Albania 4. Algeria 5. Angola 6. Antigua and Barbuda 7. Argentina 8. Armenia 9. Australia 10. Austria 11. Azerbaijan 12. Bahamas 13. Bahrain 14. Bangladesh 15. Barbados 16. Belarus 17. Belgium 18. Belize 19. Benin 20. Bhutan 21. Bosnia and Herzegovina 22. Botswana 23. Brazil 24. Brunei Darussalam 25. Bulgaria 26. Burkina Faso 27. Burundi 28. Cabo Verde 29. Cambodia 30. Cameroon 31. Canada 32. Central African Republic 33. Chad 34. Chile 35. China 36. Colombia 37. Comoros 38. Congo 39. Costa Rica 40. Croatia 41. Cuba 42. Cyprus 43. Czechia 44. Democratic People's Republic of Korea 45. Democratic Republic of the Congo 46. Denmark 47. Djibouti 48. Dominican Republic 49. Ecuador 50. Egypt 51. El Salvador 52. Equatorial Guinea 53. Eritrea 54. Estonia 55. Eswatini 56. Ethiopia 57. Fiji 58. Finland 59. France 60. Gabon 61. Gambia 62. Georgia 63. Germany 64. Ghana 65. Greece 66. Grenada 67. Guatemala 68. Guinea 69. Guinea-Bissau 70. Guyana 71. Haiti 72. Honduras 73. Hungary 74. Iceland 75. India 76. Indonesia 77. Iran (Islamic Republic of) 78. Iraq 79. Ireland 80. Israel 81. Italy 82. Jamaica 83. Japan 84. Jordan 85. Kazakhstan 86. Kenya 87. Kiribati 88. Kuwait 89. Latvia 90. Lebanon 91. Lesotho 92. Liberia 93. Libya 94. Lithuania 95. Luxembourg 96. Madagascar 97. Malawi 98. Malaysia 99. Maldives 100. Mali 101. Malta 102. Mauritania 103. Mauritius 104. Mexico 105. Micronesia (Federated States of) 106. Mongolia 107. Montenegro 108. Morocco 109. Mozambique 110. Myanmar 111. Namibia 112. Nepal 113. Netherlands 114. New Zealand 115. Nicaragua 116. Niger 117. Nigeria 118. North Macedonia 119. Norway 120. Oman 121. Pakistan 122. Panama 123. Papua New Guinea 124. Paraguay 125. Peru 126. Philippines 127. Poland 128. Portugal 129. Qatar 130. Romania 131. Russian Federation 132. Rwanda 133. Saint Lucia 134. Saint Vincent and the Grenadines 135. Samoa 136. Sao Tome and Principe 137. Saudi Arabia 138. Senegal 139. Serbia 140. Seychelles 141. Sierra Leone 142. Singapore 143. Slovenia 144. Solomon Islands 145. Somalia 146. South Africa 147. South Sudan 148. Spain 149. Sri Lanka 150. Sudan 151. Suriname 152. Sweden 153. Switzerland 154. Syrian Arab Republic 155. Tajikistan 156. Thailand 157. Timor-Leste 158. Togo 159. Tonga 160. Trinidad and Tobago 161. Tunisia 162. Turkey 163. Turkmenistan 164. Uganda 165. Ukraine 166. United Arab Emirates 167. United Kingdom of Great Britain and Northern Ireland 168. United Republic of Tanzania 169. United States of America 170. Uruguay 171. Uzbekistan 172. Vanuatu 173. Venezuela (Bolivarian Republic of) 174. Yemen 175. Zambia 176. Zimbabwe |

**S5. Comparing the regional distribution of the 18 countries excluded from the “death model” with those included in the model**

|  | Africa | Americas | Eastern Mediterranean | Europe | South-East Asia | Western Pacific |
| --- | --- | --- | --- | --- | --- | --- |
| Excluded from the model (n, %) | 0 (0) | 3 (8.6) | 0 (0) | 6 (11.5) | 0 (0) | 9 (33.3) |
| Included in the model (n, %) | 47 (100) | 32 (91.4) | 21 (100) | 47 (88.5) | 11 (100) | 18 (66.7) |

p = 0.0001 (Fisher’s test)

**S6. Comparing the capacity of the 18 countries excluded from the “death model” with those included in the model based on their capacity to manage diabetes**

|  | High capacity | Low capacity |
| --- | --- | --- |
| Excluded from the model (n, %) | 16 (9.3) | 2 (9.1) |
| Included in the model (n, %) | 156 (90.7) | 20 (90.9) |

p = 0.974, *χ*2=0.001
